# Sex differences in early and late neonatal all-cause mortality and disease burden in Kazakhstan, 2010–2023: A population-based analysis of Global Burden of Disease estimates

**DOI:** 10.1371/journal.pone.0347911

**Published:** 2026-10-08

**Authors:** Yelena Tsay, Bekzhan Namet, Saltanat Urazova, Nazym Galimgozhina, Lyailyum Saparova, Nazym Amanbayeva, Madina Syzdykova, Yuliya Ganzhula, Isida Ashirmatova, Gulnur Ashirova, Indira Karibayeva

**Affiliations:** 1 Department of Family Medicine No. 3, Astana Medical University, Astana, Kazakhstan; 2 Department of Children’s Diseases No. 1, Astana Medical University, Astana, Kazakhstan; 3 Department of Health Policy and Community Health, Jiann-Ping Hsu College of Public Health, Georgia Southern University, Statesboro, Georgia, United States of America; 4 Department of Research Management, JSC Research Institute of Cardiology and Internal Diseases, Almaty, Kazakhstan; Isfahan University of Medical Sciences, IRAN, ISLAMIC REPUBLIC OF

## Abstract

**Background:**

Neonatal mortality remains a major contributor to child mortality globally, with the greatest burden concentrated in the early neonatal period. Sex differences in neonatal survival are consistently reported, yet population-level evidence on sex-specific patterns of all-cause burden across neonatal subperiods in Central Asia remains limited. This study aimed to quantify temporal trends and sex differences in early (0–6 days) and late (7–27 days) neonatal all-cause mortality and disease burden in Kazakhstan from 2010 to 2023, and to examine projected trajectories to 2030.

**Methods:**

We conducted a population-based time-trend study using Global Burden of Disease 2023 estimates for Kazakhstan. Outcomes included mortality, disability-adjusted life years (DALYs), years of life lost (YLLs), and years lived with disability (YLDs), expressed as age-specific rates per 100,000 person-years within the corresponding neonatal age interval. Analyses were stratified by sex and neonatal subperiod. Temporal patterns were described, and sex differences were assessed using comparative rates. Log-linear models were used to estimate projected trends to 2030. National mortality statistics were used for external trend consistency.

**Results:**

Neonatal mortality and DALY rates declined between 2010 and 2023 in both early and late neonatal periods, with substantially higher rates observed in the early neonatal period. Across all years, males consistently exhibited higher mortality, YLLs, and DALYs than females in both neonatal subperiods; YLD rates followed a different pattern, being modestly higher among females in the early-neonatal period but nearly equal between sexes in the late-neonatal period. YLLs accounted for the vast majority of DALYs, indicating that mortality rather than non-fatal disability drives neonatal burden. Projections suggest continued declines through 2030, male point estimates for mortality and DALYs remained higher than female estimates throughout, although the magnitude of this difference narrowed substantially and became statistically uncertain in the most recent years, with rate-ratio uncertainty intervals spanning or approaching unity.

**Conclusions:**

Despite substantial reductions in neonatal mortality in Kazakhstan, male point estimates for mortality and burden remained higher than female estimates throughout the study period, although this difference narrowed substantially and became uncertain in recent years; mortality remained heavily concentrated in the early neonatal period. These findings highlight persistent, though narrowing, epidemiologic inequalities within improving population health trends.

## 1. Introduction

Neonatal mortality and disability remain fundamental indicators of health system performance, as survival and healthy development in the first month of life depend on timely, continuous, and high‑quality care spanning pregnancy, childbirth, the immediate postnatal period, and early infancy. From a theoretical standpoint, the WHO Health Systems Framework positions neonatal outcomes as a reflection of system performance across its core building blocks—service delivery, health workforce, financing, governance, information systems, and access to essential medicines [1]. At the same time, the Life Course perspective underscores the neonatal period as a critical developmental window, where early disadvantage can accumulate into long‑term health, educational, and socioeconomic inequities [2]. Together, these frameworks highlight neonatal survival not only as a measure of immediate system effectiveness but also as a predictor of population well‑being across the lifespan.

Despite substantial global improvements in neonatal outcomes over recent decades, progress has been uneven across countries and regions, with preventable causes—such as complications of prematurity, neonatal infections, and acute respiratory or gastrointestinal conditions—continuing to drive early life losses [3]. UNICEF estimates that 2.3 million infants died during the neonatal period in 2023, equivalent to roughly 6,300 deaths each day worldwide [4]. Current projections suggest that between 2020 and 2030, approximately 48 million deaths among children under five could occur, with nearly half expected during the neonatal period [5]. In middle‑income settings, persistent gaps often reflect barriers in early identification of risk, delayed care seeking, discontinuities between facility and community services, and variable access to responsive primary health care [6]. Moreover, sustained socioeconomic hardship and psychosocial adversity in the earliest years of life can produce long‑lasting harms, including worse health, lower educational attainment, and reduced adult income, underscoring early childhood as a critical window for prevention [7]. Families with fewer socioeconomic resources often face greater obstacles to accessing health and social support services, magnifying developmental risks for children.

Kazakhstan, as an upper-middle-income country in Central Asia [8], has experienced notable improvements in maternal and child health over the past two decades. Neonatal mortality has declined in parallel with broader health system strengthening, increased access to skilled birth attendance, and improvements in perinatal and neonatal care [9,10]. However, neonatal outcomes remain a key component of national health priorities, particularly given persistent regional disparities and the relatively high contribution of neonatal deaths to overall child mortality. Previous national and regional studies have primarily focused on mortality indicators, with limited attention to the broader burden of disease, including disability and long-term health consequences. Furthermore, there is limited evidence on future trajectories of neonatal burden in Kazakhstan, which is essential for informing policy planning and resource allocation.

To address this gap, this study aimed to quantify temporal trends and sex differences in early and late neonatal mortality and disease burden in Kazakhstan from 2010 to 2023 using Global Burden of Disease 2023 estimates. In addition, we examined the relative contribution of fatal and non-fatal components of burden and explored projected trajectories to 2030.

Most existing GBD-based analyses of neonatal mortality report all-age neonatal burden or focus on single-country settings outside Central Asia; sex-specific stratification by early- and late-neonatal subperiod, combined with disaggregation of fatal and non-fatal burden and forward projection to 2030, has not previously been reported for Kazakhstan or, to our knowledge, for a Central Asian country. Kazakhstan is a useful setting for this analysis because it combines upper-middle-income status, rapid recent health-system reform, and a relatively data-complete vital registration system feeding into GBD estimation, making it a plausible case for observing how sex-specific neonatal disparities evolve alongside health-system strengthening in a transitional-economy context underrepresented in the neonatal GBD literature.

## 2. Methods

### 2.1. Study design, data source, and study outcomes

This study was a population-level descriptive and modeling analysis of neonatal mortality and disease burden in Kazakhstan using estimates from the Global Burden of Disease (GBD) 2023 study. The GBD study provides comprehensive and standardized estimates of mortality and morbidity for a wide range of diseases and injuries across countries and over time using a combination of epidemiological data sources, statistical modeling, and comparative risk assessment frameworks. The detailed description of the GBD methods for data collection and estimation process is provided elsewhere [11–13]. The GBD framework generates internally consistent estimates of mortality and other health indicators by age, sex, location, and year using a combination of primary data sources, statistical modeling, and cause-of-death ensemble methods. Information on all data inputs used in generating GBD 2023 estimates is available through the Global Health Data Exchange (GHDx) Sources Tool for GBD 2023 [14].

For this study, data specific to Kazakhstan were extracted from the Global Burden of Disease 2023 database for the period 2010–2023. The data were accessed for research purposes on 19 January 2026. Neonatal mortality was defined according to GBD age classifications and disaggregated into early neonatal (0–6 days) and late neonatal (7–27 days) periods. Estimates were retrieved separately for males and females to enable sex-specific analyses, as well as for both sexes combined where appropriate. GBD age-specific rates are expressed per 100,000 person-years within the early-neonatal (0–6 days) or late-neonatal (7–27 days) age interval and should not be interpreted as mortality probabilities per live birth. Data were extracted from the GBD 2023 Results Tool for Kazakhstan (location_id 36), cause “All causes” (cause_id 294), sex Male/Female/Both (sex_id 1/2/3), ages Early Neonatal (0–6 days, age_id 2) and Late Neonatal (7–27 days, age_id 3), measures Deaths, DALYs, YLLs, and YLDs (measure_id 1/2/4/3), metric Rate (metric_id 3), for years 2010–2023. All-cause burden was extracted directly as the pre-aggregated “All causes” GBD category rather than calculated by summing individual causes. The authors did not have access to any information that could identify individual participants during or after data extraction, as the study used only aggregated, de-identified secondary data.

GBD 2023 produces estimates across a comprehensive set of diseases, injuries, and risk factors, stratified by age group, sex, geographic location, and year [11–13]. These estimates are derived using standardized modeling approaches that integrate multiple data sources, including vital registration systems, surveys, and surveillance data, to ensure comparability across settings. For Kazakhstan, neonatal mortality estimates therefore reflect modeled outputs that account for data completeness, potential misclassification, and temporal trends, enabling consistent comparisons over time and between population subgroups.

The primary outcomes of interest were all-cause neonatal mortality and disease burden, assessed using four standard GBD health metrics: Deaths, Disability-adjusted life years (DALYs), Years of life lost due to premature mortality (YLLs), Years lived with disability (YLDs).

DALYs represent the sum of fatal and non-fatal health loss and are defined as the combination of YLLs and YLDs. These metrics allow quantification of both mortality and morbidity components of neonatal health burden.

For the primary analysis, we focused on all-cause neonatal burden, which captures the aggregate health loss across all neonatal conditions. Uncertainty intervals provided by the GBD study (lower and upper bounds) were incorporated into descriptive visualizations to illustrate the uncertainty surrounding modeled estimates.

To provide additional context for the findings, we incorporated an external trend comparison using officially reported national mortality indicators. Specifically, we used the indicator “infant mortality rate in the Republic of Kazakhstan per 1,000 live births” obtained from national statistical sources for the same study period [15]. Given differences in age definitions (infant vs. neonatal), denominators, and estimation methodologies between GBD outputs and national statistics, direct numerical equivalence between these measures was not expected. Because GBD estimates themselves incorporate national vital-registration and government data as inputs, the national statistics used here are not fully independent of the GBD estimation process; accordingly, this comparison is presented as a trend concordance check rather than an external validation, and does not on its own establish that GBD-derived estimates are free of modeling artifacts.

In addition, we examined male infant mortality rates per 1,000 live births derived from national data to assess whether the sex-specific patterns identified in the GBD neonatal analysis were also reflected in routinely collected national infant mortality statistics, while recognizing that infant and neonatal mortality are not the same measure (see Limitations).

Ethical approval for this study was waived by the Local Bioethics Committee of Astana Medical University, as the analysis was based exclusively on secondary, aggregated data obtained from publicly available sources and did not involve identifiable human participants. Patient consent was not applicable, as the study did not involve identifiable individual participants.

### 2.2. Statistical analysis and modeling description

Analyses for original GBD 2023 were implemented in Python, Stata, and R, with full code made publicly available [16]. Analyses for the present study were conducted using R version 4.5.1 (2025-06-13) [17] within the RStudio environment (version 2025.9.0.387) [18]. Descriptive analyses summarized annual estimates of deaths, DALYs, YLLs, and YLDs by sex and neonatal age group. Temporal patterns were visualized using line plots with uncertainty ribbons representing the GBD-provided lower and upper bounds of the estimates.

To quantify temporal changes in neonatal burden, we evaluated trends in each outcome measure between 2010 and 2023. Percentage change between the first and last year of observation was calculated for each outcome, sex, and neonatal age group. In addition, estimated annual percent change (EAPC) was calculated using log-linear regression models of the form:


$$\begin{array}{c} \text{log}\left(rate_{t}\right)=\alpha +\beta \times year_{t} \end{array}$$

where $rate_{t}$ represents the annual rate per 100,000 person-year in year t. The estimated annual percent change was derived as:


$$\begin{array}{c} EAPC=\text{1}\text{0}\text{0}\times \left(e^{\beta }-\text{1}\right) \end{array}$$

This approach allows consistent comparison of temporal trends across outcomes and demographic strata.

Sex differences were assessed by comparing rates between males and females over time and across neonatal age groups. Differences were visualized using sex-specific trend plots and male-to-female comparisons in projected estimates.

Male-to-female rate ratios (RRs) were calculated by dividing the male rate by the corresponding female rate for each year, outcome, and neonatal subperiod. Because GBD posterior draws were not available, approximate 95% uncertainty intervals for the RRs were derived from the reported sex-specific GBD uncertainty intervals using a delta-method approximation on the log scale. This calculation necessarily assumes independence between the male and female estimates because their joint covariance was unavailable. The resulting intervals should therefore be regarded as approximate and do not reproduce draw-level GBD uncertainty.

GBD uncertainty intervals were incorporated into descriptive visualizations only (Figs 1–4) and were not propagated into the EAPC or forecasting models, which were fitted to annual point estimates; because posterior draw-level data were not available to the authors, the reported forecasting intervals reflect only sampling uncertainty in the fitted trend and likely understate the true uncertainty of the projections (see Limitations). To estimate future neonatal burden in Kazakhstan, projections of deaths and DALYs were generated through 2030 using log-linear regression models fitted to observed rates from 2010–2023. For each outcome, age group, and sex, a regression model was fitted to the natural logarithm of the rate as a function of calendar year. Predicted rates for future years were then obtained by exponentiating the fitted values. For each outcome, age group, and sex, 95% prediction intervals for the year-2030 forecast were obtained from the fitted regression model (predict(..., interval = “prediction”)), reflecting uncertainty in a new observation around the fitted trend rather than uncertainty in the mean trend itself. Residual diagnostics (Durbin-Watson and Breusch-Godfrey tests) indicated significant positive autocorrelation in the residuals of most log-linear models, particularly in the early-neonatal period, confirming that these are correlated time-series observations rather than independent draws and that the reported prediction intervals, which assume independent errors, likely understate the true forecast uncertainty. The same log-linear models underlie the EAPC estimates reported in the Results, and the segmented-regression model described below likewise assumes independent residuals when computing the Davies’ test p-value; given the autocorrelation documented here, the 95% CIs and p-values associated with the EAPC and the segmented-regression breakpoint should be interpreted as nominal rather than autocorrelation-robust statistics, and are likely anti-conservative.

**Fig 1:**
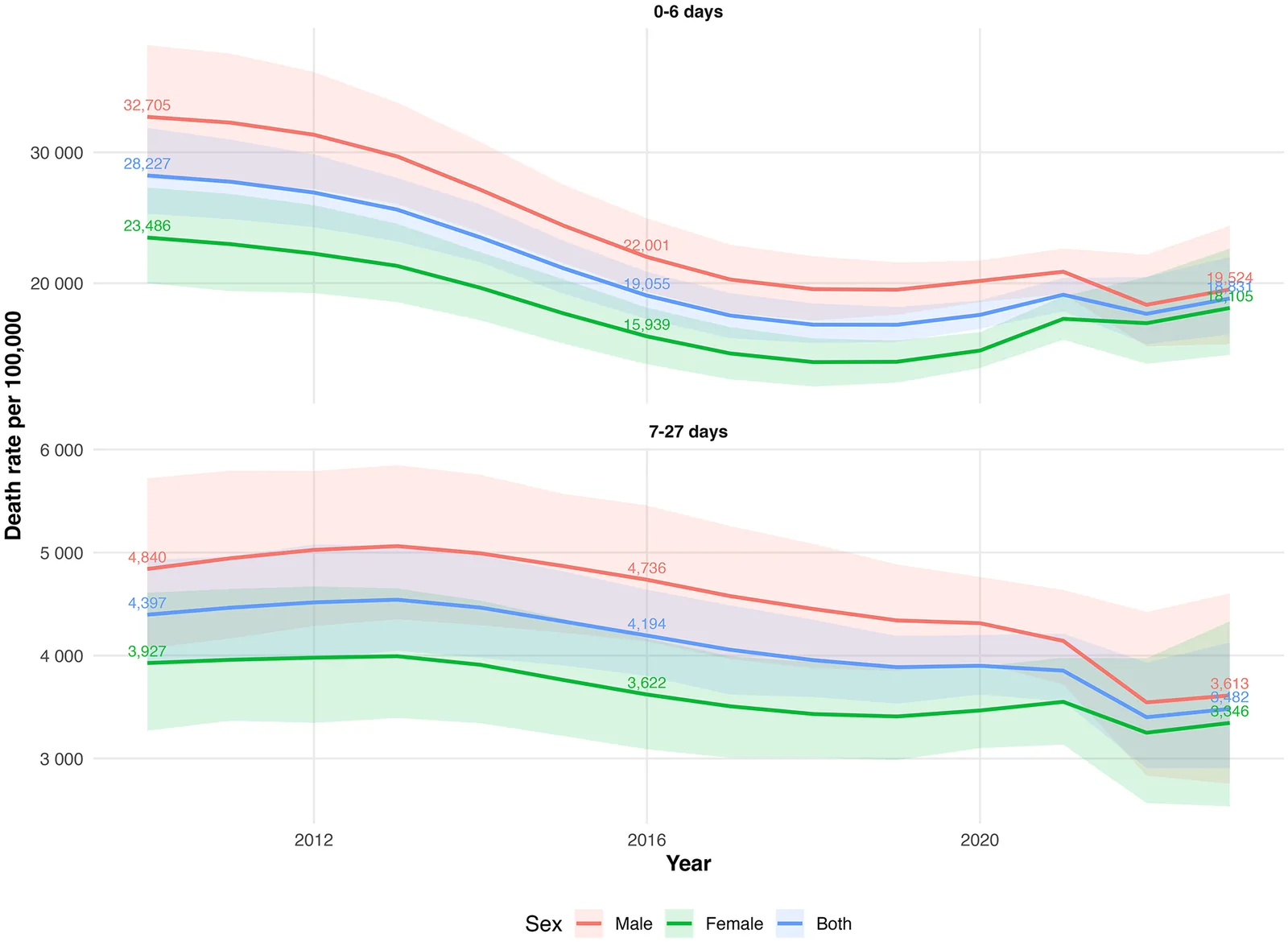
Trends in all-cause neonatal mortality rates in Kazakhstan, 2010–2023. Note: GBD age-specific rates are expressed per 100,000 person-years within the specified neonatal age interval and are not mortality probabilities per live birth. Shading indicates 95% GBD uncertainty intervals.

**Fig 2:**
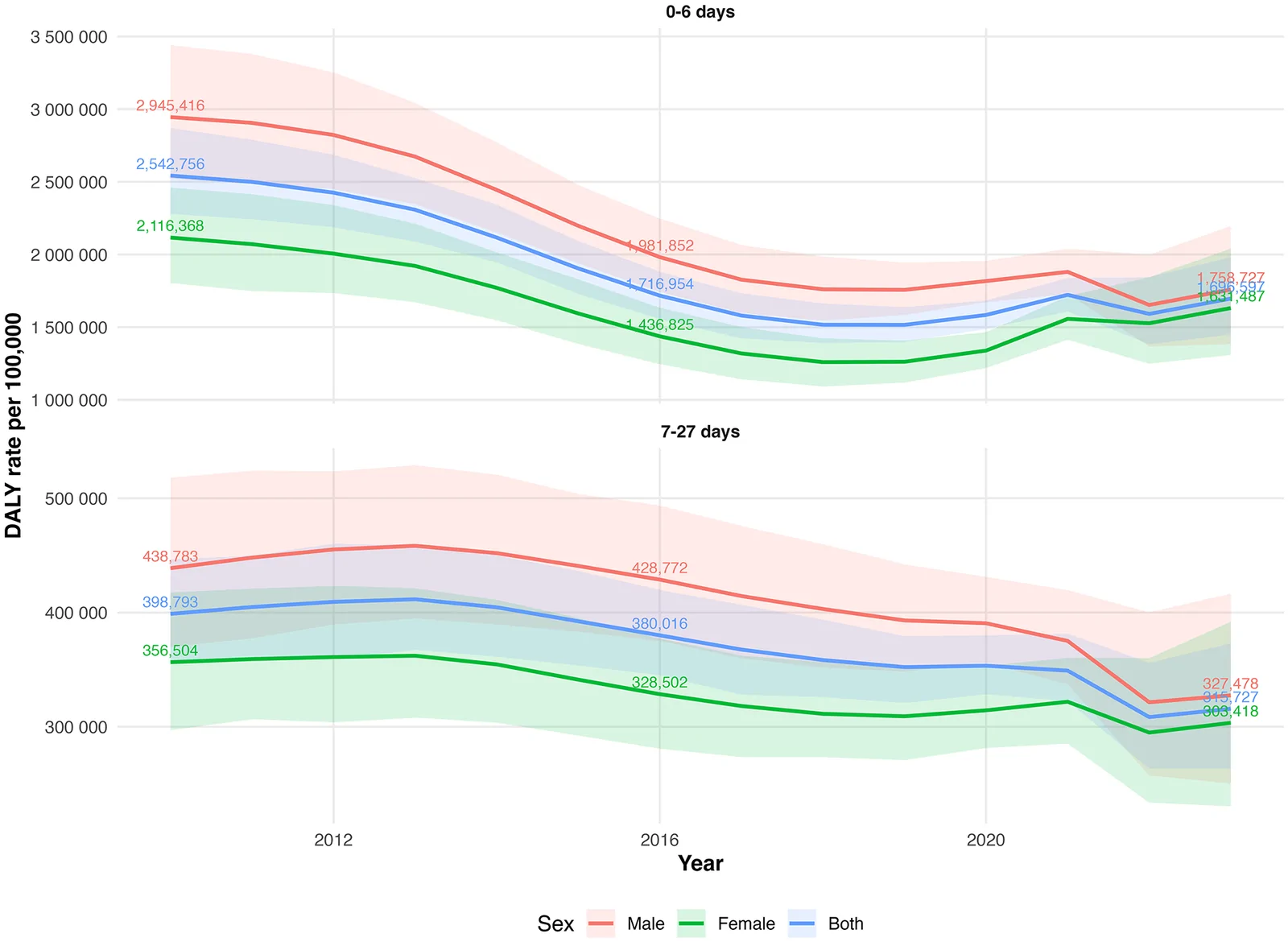
Trends in all-cause neonatal DALY rate in Kazakhstan, 2010–2023. Note. GBD age-specific rates are expressed per 100,000 person-years within the specified neonatal age interval and are not mortality probabilities per live birth. Shading indicates 95% GBD uncertainty intervals.

**Fig 3:**
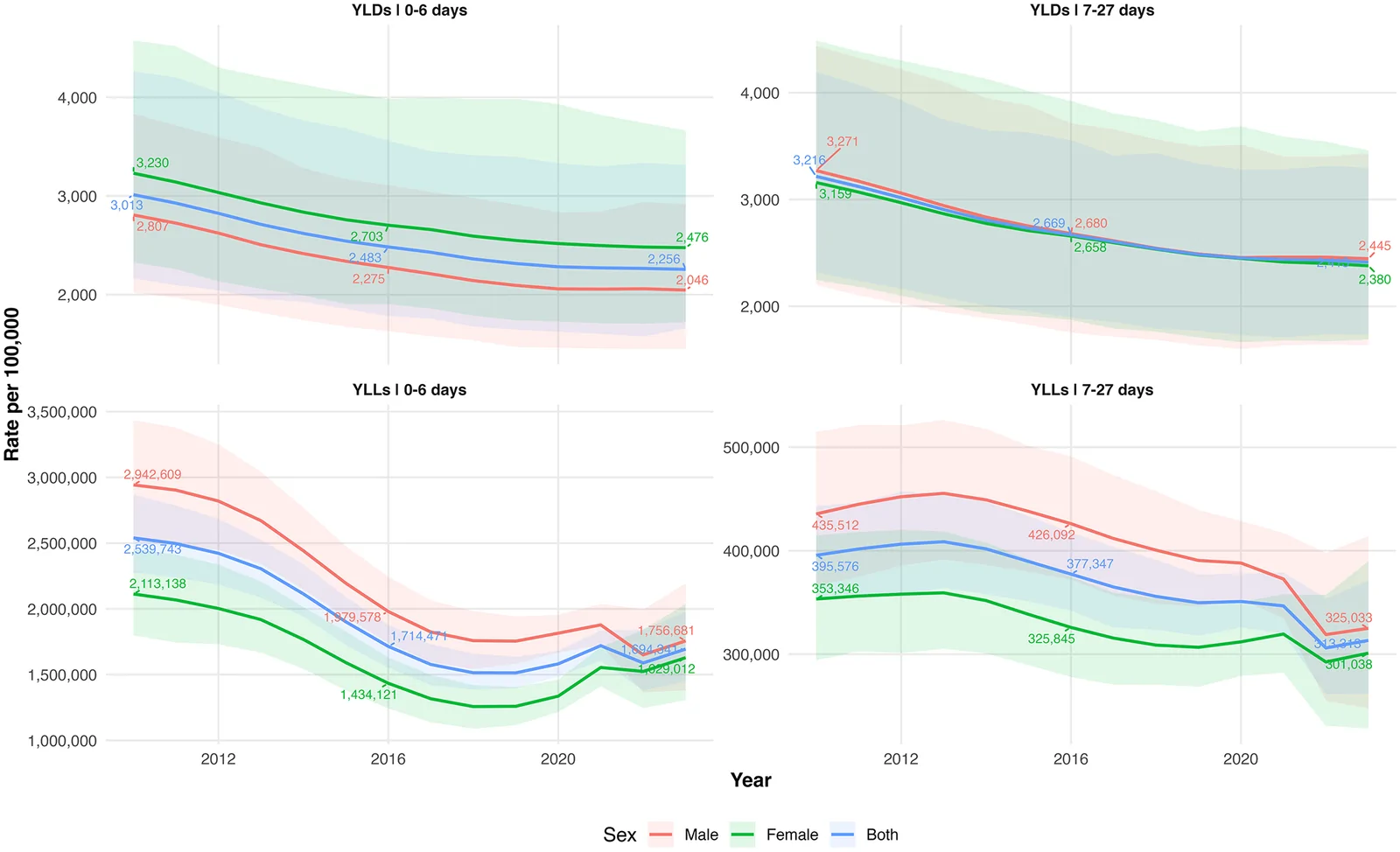
Mortality and disability components of neonatal burden in Kazakhstan, 2010-2023. Note. GBD age-specific rates are expressed per 100,000 person-years within the specified neonatal age interval and are not mortality probabilities per live birth. Shading indicates 95% GBD uncertainty intervals.

**Fig 4:**
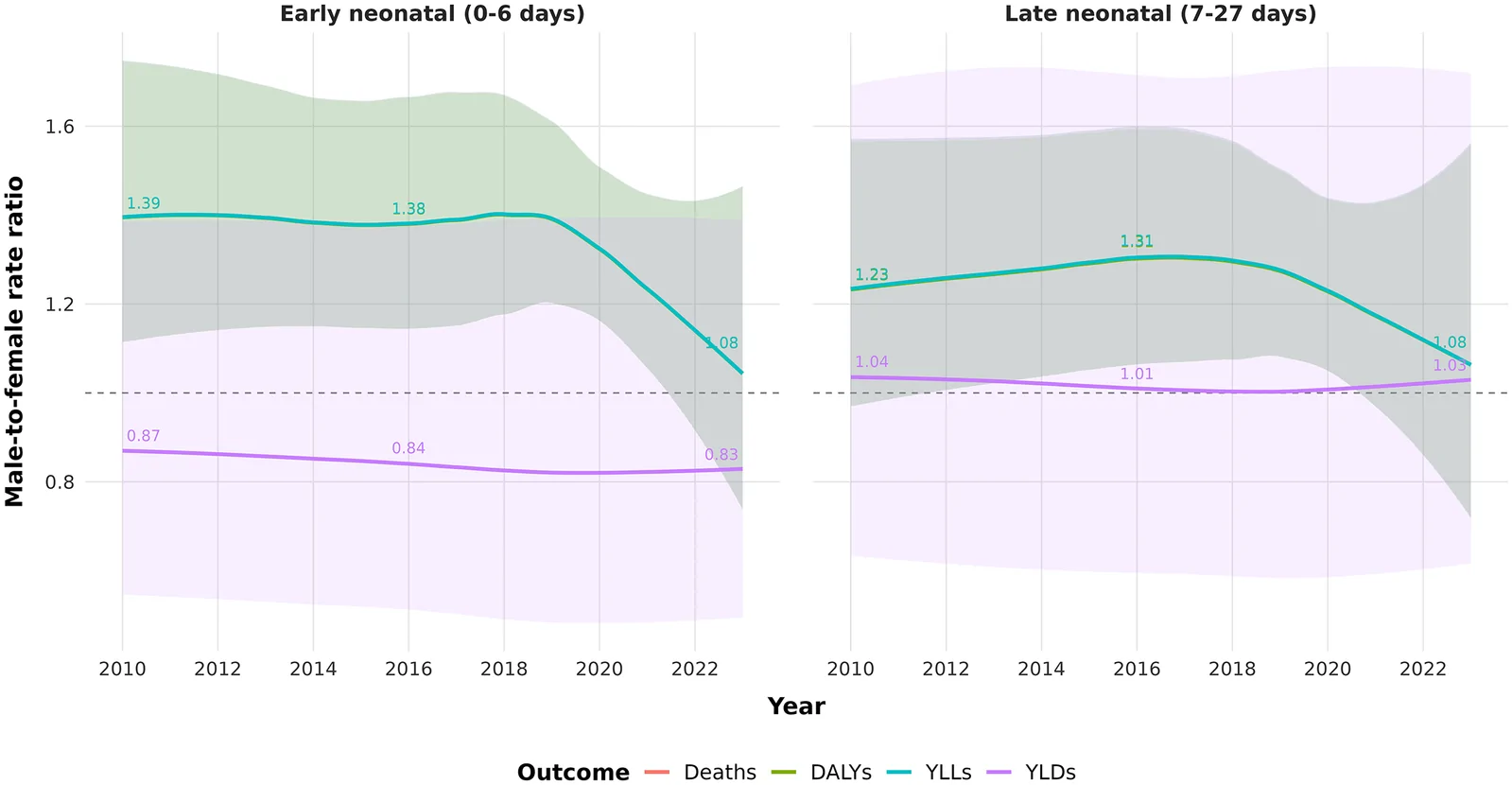
Male-to-female rate ratios for neonatal burden in Kazakhstan, 2010-2023. Note. Approximate 95% UI’s (shaded) computed by the delta method assuming independence between sex-specific GBD estimates.

The log-linear model was retained as the primary forecasting approach because it assumes proportional change in rates over time, yields an interpretable constant proportional annual-change parameter (EAPC), and has been widely used in comparable GBD-based trend studies. To formally test whether a single constant rate of change adequately describes the observed trend, we additionally fitted a segmented (broken-stick) regression to the early-neonatal log(rate) series for Deaths and DALYs (both sexes combined) and tested for a non-constant slope using Davies’ test.

To evaluate the robustness of projected estimates, two additional forecasting approaches were implemented as sensitivity analyses: 1) Linear regression models on the original rate scale, which assume constant absolute change over time. 2) Exponential smoothing time-series models (ETS), which allow data-driven estimation of trend patterns without imposing a predefined functional form. For each series, the ETS model form (error, trend, and seasonal components) was selected automatically by minimizing AICc using the ets() function in the R forecast package; the selected form is reported for each series in the Results. Projected values for 2030 were compared across models using a three-year rolling-origin out-of-sample root-mean-square error (RMSE; holdout years 2021–2023) as the primary, scale-consistent metric of forecast performance; in-sample AIC/BIC are also reported for the two regression models but are not comparable across response scales (log vs. original) or to ETS.

Because the GBD early- and late-neonatal rates are age-specific person-time rates with different denominators, they were not combined into a single 0–27-day rate. Instead, each GBD age-specific mortality series was compared separately with the national infant mortality rate. Descriptive concordance was assessed using Pearson and Spearman correlations for annual levels, Pearson correlations for first differences, and the proportion of consecutive year-pairs showing changes in the same direction. These comparisons were intended to assess broad temporal concordance rather than numerical agreement or external validation.

### 2.3. Use of generative AI tools

Generative AI (ChatGPT, OpenAI) was used solely for language editing of the manuscript, including suggestions related to grammar, sentence structure, readability, and clarity. It was not used to generate or analyze study data, perform statistical analyses, select analytical methods, generate numerical results, create figures or tables, or prepare the underlying dataset or analytical code. All AI-generated language suggestions were critically reviewed against the underlying analyses and source material and were accepted, modified, or rejected by the authors as appropriate. The authors take full responsibility for the accuracy and integrity of the final manuscript.

## 3. Results

Neonatal mortality rates declined in Kazakhstan between 2010 and 2023 in both early and late neonatal periods, with consistently higher mortality observed in the early neonatal period across all years. Across the study period, males exhibited higher mortality rates than females in both neonatal subperiods. The male excess was persistent for mortality, YLLs, and DALYs, whereas YLD patterns differed by neonatal subperiod. Fig 1 presents trends in all-cause neonatal mortality rates in Kazakhstan from 2010 to 2023, stratified by neonatal age group and sex. Overall, neonatal mortality declined substantially during the study period in both early neonatal (0–6 days) and late neonatal (7–27 days) populations, although reductions were more pronounced during the early neonatal period. In 2010, the early neonatal (0–6 days) mortality rate was 32,705 deaths per 100,000 person-years among males and 23,486 per 100,000 person-years among females, corresponding to a combined rate of 28,227 per 100,000 person-years (95% UI 25,303–31,856; Table 1). Mortality declined steadily over the following decade, reaching 19,524 per 100,000 person-years among males and 18,105 per 100,000 person-years among females by 2023. The combined early neonatal mortality rate decreased to 18,831 per 100,000 person-years, representing an overall decline of approximately 33% compared with 2010 levels (EAPC –3.96% per year, 95% CI –5.39 to –2.51; Table 2). Mortality during the late neonatal period (7–27 days) was substantially lower than in the early neonatal stage but also declined over time. In 2010, late neonatal mortality rates were 4,840 deaths per 100,000 person-years among males and 3,927 per 100,000 person-years among females, corresponding to a combined rate of 4,397 per 100,000 person-years (95% UI 3,913–4,927; Table 1). By 2023, these values had declined to 3,613 per 100,000 person-years among males and 3,346 per 100,000 person-years among females. The combined late neonatal mortality rate reached 3,482 per 100,000 person-years, corresponding to an approximate 21% reduction from 2010 levels (EAPC –2.07% per year, 95% CI –2.57 to –1.56; Table 2).

**Table 1:** GBD 2023 all-cause neonatal mortality and disease burden rates in Kazakhstan, by neonatal period and sex, 2010 and 2023.

Measure	Sex	Rate per 100,000 person-years within age interval (95% UI)		Change 2010−23, %
		2010	2023	
**Early neonatal (0–6 days)**				
**Deaths**	Male	32,705 (27,966−38,180)	19,524 (15,357−24,388)	−40.3
	Female	23,486 (20,001-27,303)	18,105 (14,526−22,641)	−22.9
	Both sexes	28,227 (25,303−31,856)	18,831 (16,086−22,004)	−33.3
**DALYs**	Male	2,945,416 (2,519,421−3,439,700)	1,758,727 (1,384,428−2,195,902)	−40.3
	Female	2,116,368 (1,803,638−2,459,879)	1,631,487 (1,309,134−2,039,918)	−22.9
	Both sexes	2,542,756 (2,279,678−2,870,124)	1,696,597 (1,449,393−1,982,452)	−33.3
**YLLs**	Male	2,942,609 (2,516,240−3,435,262)	1,756,681 (1,381,785−2,194,281)	−40.3
	Female	2,113,138 (1,799,557−2,456,603)	1,629,012 (1,306,988−2,037,125)	−22.9
	Both sexes	2,539,743 (2,276,684−2,866,241)	1,694,341 (1,447,331−1,979,816)	−33.3
**YLDs**	Male	2,807 (2,020 3,831)	2,046 (1,450−2,916)	−27.1
	Female	3,230 (2,324−4,576)	2,476 (1,723−3,665)	−23.4
	Both sexes	3,013 (2,164−4,262)	2,256 (1,659−3,313)	−25.1
**Late neonatal (7–27 days)**				
**Deaths**	Male	4,840 (4,069−5,721)	3,613 (2,754−4,603)	−25.4
	Female	3,927 (3,271−4,610)	3,346 (2,535−4,332)	−14.8
	Both sexes	4,397 (3,913−4,927)	3,482 (2,904−4,127)	−20.8
**DALYs**	Male	438,783 (369,871−518,050)	327,478 (250,352-416,485)	−25.4
	Female	356,504 (297,101-417,578)	303,418 (230,303-391,816)	−14.9
	Both sexes	398,793 (355,186-446,422)	315,727 (263,376−373,036)	−20.8
**YLLs**	Male	435,512 (366,138-514,707)	325,033 (247,815−414,146)	−25.4
	Female	353,346 (294,289-414,780)	301,038 (228,111-389,751)	−14.8
	Both sexes	395,576 (352,035-443,319)	313,313 (261,290-371,303)	−20.8
**YLDs**	Male	3,271 (2,199−4,439)	2,445 (1,632−3,433)	−25.2
	Female	3,159 (2,245−4,489)	2,380 (1,690−3,461)	−24.7
	Both sexes	3,216 (2,316−4,195)	2,413 (1,728−3,299)	−25.0

Note. Values in parentheses are 95% uncertainty intervals (UI).

Percentage change=((2023 estimate - 2010 estimate)/ 2010 estimate) x 100; negative values indicate reductions.

Abbreviations: DALYs = disability-adjusted life-years. YLLs = years of life lost. YLDs = years lived with disability. GBD = Global Burden of Disease.

**Table 2:** Estimated annual percentage change in all-cause neonatal mortality and disease burden rates in Kazakhstan, 2010-2023.

Measure	Sex	EAPC, % (95% CI)	*p* value
**Early neonatal (0–6 days)**			
Deaths	Male	−4.65 (−5.77 to −3.52)	<0.001
	Female	−3.04 (−4.92 to −1.12)	0.005
	Both sexes	−3.96 (−5.39 to −2.51)	<0.001
DALYs	Male	−4.65 (−5.77 to −3.52)	<0.001
	Female	−3.04 (−4.92 to −1.12)	0.005
	Both sexes	−3.96 (−5.38 to −2.51)	<0.001
YLLs	Male	−4.65 (−5.77 to −3.52)	<0.001
	Female	−3.04 (−4.92 to −1.12)	0.005
	Both sexes	−3.96 (−5.39 to −2.51)	<0.001
YLDs	Male	−2.55 (−2.96 to −2.15)	<0.001
	Female	−2.09 (−2.40 to −1.78)	<0.001
	Both sexes	−2.30 (−2.66 to −1.95)	<0.001
**Late neonatal (7–27 days)**			
Deaths	Male	−2.46 (−3.21 to −1.70)	<0.001
	Female	−1.58 (−1.99 to −1.18)	<0.001
	Both sexes	−2.07 (−2.57 to −1.56)	<0.001
DALYs	Male	−2.46 (−3.20 to −1.71)	<0.001
	Female	−1.59 (−1.99 to −1.19)	<0.001
	Both sexes	−2.07 (−2.57 to −1.57)	<0.001
YLLs	Male	−2.46 (−3.21 to −1.70)	<0.001
	Female	−1.58 (−1.99 to −1.18)	<0.001
	Both sexes	−2.07 (−2.57 to −1.56)	<0.001
YLDs	Male	−2.33 (−2.72 to −1.93)	<0.001
	Female	−2.20 (−2.46 to −1.94)	<0.001
	Both sexes	−2.27 (−2.59 to −1.94)	<0.001

Note. EAPC estimates were derived from log-linear regression of log(rate) on calendar year and are presented with 95% confidence intervals (CI). EAPC = 100 x [exp(beta) – 1], where beta is the regression slope; negative values indicate an average annual decline. *p* values correspond to the regression slope term. Because the underlying log-linear models showed significant residual autocorrelation (Section 2.2, Table S3 in S1 File), these CIs and p values assume independent residuals and likely understate the true uncertainty of the EAPC estimates.

Abbreviations: DALYs = disability-adjusted life-years. YLLs = years of life lost. YLDs = years lived with disability. EAPC = estimated annual percentage change.

Trends in all-cause DALY rates are presented in Fig 2. Consistent with the mortality trends, the overall disease burden during the early neonatal period also declined substantially. In 2010, the DALY rate was 2,945,416 per 100,000 person-years among males and 2,116,368 among females (95% UI 1,803,638–2,459,879; Table 1). By 2023, these values had declined to 1,758,727 per 100,000 person-years among males and 1,631,487 among females (95% UI 1,309,134–2,039,918; Table 1). The combined DALY rate decreased from 2,542,756 per 100,000 person-years in 2010–1,696,597 per 100,000 person-years in 2023 (EAPC for DALYs, both sexes: –3.96% per year early-neonatal, –2.07% per year late-neonatal; Table 2). The early neonatal burden was overwhelmingly driven by YLL rather than disability. In 2010, YLL rates reached 2,942,609 per 100,000 person-years among males and 2,113,138 among females, whereas YLD rates were substantially lower at 2,807 and 3,230 per 100,000 person-years, respectively. A similar pattern persisted in 2023, with YLL rates remaining orders of magnitude higher than YLD rates. Across all years, male mortality rates consistently exceeded female mortality, indicating a persistent sex disparity in early neonatal survival, although the magnitude of this disparity narrowed substantially over the study period (see Table S1 in S1 File). Patterns of disease burden during the late neonatal period were similar to those observed in the early neonatal stage but at substantially lower levels. DALY rates decreased from 438,783 per 100,000 person-years among males in 2010–327,478 in 2023, while female DALY rates declined from 356,504–303,418 per 100,000 person-years. As in the early neonatal period, the majority of DALYs were attributable to premature mortality (YLLs) rather than disability.

During early neonatal period YLL rates declined markedly over the study period for all sex groups. Among males, YLLs decreased from approximately 2,942,609 per 100,000 person-years in 2010–1,756,681 in 2023, representing a reduction of roughly 40%. A similar pattern was observed among females, with YLL rates declining from 2,113,138–1,629,012 per 100,000 person-years, while the combined population decreased from 2,539,743–1,694,341 per 100,000 person-years. The decline was most pronounced between 2010 and 2016, after which rates stabilized and showed a modest increase around 2020–2021, followed by a subsequent decline toward 2023. Segmented regression of the both-sex early-neonatal mortality and DALY series identified a change in trend around 2018 (Davies’ test p < 0.001, assuming independent residuals; Table S2 in S1 File), with an average annual decline of approximately 7% before the breakpoint followed by a flat to slightly increasing trend of approximately 2% per year during 2018–2023; because the underlying regression residuals showed significant autocorrelation (Table S3 in S1 File), this p-value likely understates the true uncertainty of the breakpoint and should be interpreted as nominal rather than autocorrelation-robust (see Section 2.2). In contrast, YLD rates were substantially lower and declined more gradually. Male YLD rates decreased from approximately 2,807 per 100,000 person-years in 2010–2,046 in 2023, while female YLDs declined from 3,230–2,476 per 100,000 person-years. The combined YLD rate decreased from 3,013–2,256 per 100,000 person-years over the same period. In the early-neonatal period, female YLD rates consistently exceeded male rates, in contrast to YLL patterns. Late neonatal YLL rates were substantially lower than those observed in the early neonatal period but followed a similar declining trajectory. Among males, YLL rates decreased from approximately 435,512 per 100,000 person-years in 2010–325,033 in 2023, while female YLLs declined from 353,346–301,038 per 100,000 person-years. The combined YLL rate decreased from 395,576–313,313 per 100,000 person-years. YLD rates in the late neonatal period were again minimal relative to YLLs. Male YLDs declined from approximately 3,271 per 100,000 person-years in 2010–2,445 in 2023, and female YLDs decreased from 3,159–2,380 per 100,000 person-years, with the combined rate falling from 3,216–2,413 per 100,000 person-years — male and female late-neonatal YLD rates were nearly identical throughout, unlike the clearer female excess seen in the early-neonatal period.

Fig 4 presents male-to-female RRs for neonatal mortality and disease burden in Kazakhstan from 2010 to 2023, stratified by age group and outcome, together with approximate 95% uncertainty intervals computed by the delta method from the GBD-reported male and female uncertainty intervals (Table S1 in S1 File). A rate ratio greater than 1 indicates a higher burden among males. In the early neonatal period, male point estimates exceeded female point estimates across all mortality-related outcomes. For DALYs, the male-to-female RR remained elevated throughout the study period, fluctuating around 1.35–1.40 between 2010 and 2019 (95% UI excluding 1 in these years), before declining sharply to approximately 1.08 by 2022–2023, with the approximate 95% UI in the most recent years spanning or closely approaching 1. A similar pattern was observed for mortality and YLLs, which closely mirrored the DALY pattern given the dominant contribution of mortality to overall burden, declining from approximately 1.35–1.40 to approximately 1.08–1.10 by 2023. In contrast, early-neonatal YLDs exhibited an inverse pattern, with RRs consistently below 1 throughout the study period (approximately 0.87 in 2010 declining modestly to 0.82–0.84 in later years), indicating a modestly higher disability burden among females, with relatively narrow temporal variation compared to mortality-related measures. Sex disparities in the late neonatal period were less pronounced but followed a similar overall trajectory for mortality-related outcomes: the DALY RR rose from approximately 1.23 in 2010 to 1.30 around 2016 before declining to approximately 1.08 by 2023, with mortality and YLL RRs following a comparable path. In contrast, YLD RRs in the late neonatal period were close to unity throughout the study period, fluctuating around 1.00–1.05 and, in most years, marginally favoring a small male excess rather than the female excess seen in the early-neonatal period — the reverse pattern from early-neonatal YLDs.

Fig 5 presents projected trends in neonatal mortality and disease burden in Kazakhstan through 2030 based on log-linear models fitted to 2010–2023 data. Across both neonatal age groups and outcomes, projections indicate continued declines in mortality and DALY rates, with reductions occurring across all sex groups. In the early neonatal period, DALY rates are projected to decline substantially by 2030. Among males, DALYs are projected to decrease to approximately 1,117,929 per 100,000 person-years, while rates among females are projected to reach 1,057,849 per 100,000 person-years. The combined population is projected to decline to approximately 1,086,312 per 100,000 person-years (95% prediction interval 797,867–1,479,037; Table 3), reflecting a continued downward trajectory consistent with historical trends. Similarly, early neonatal mortality is projected to decrease across all sex groups. Male mortality is projected to decline to approximately 12,407 deaths per 100,000 person-years by 2030, compared with 11,734 per 100,000 person-years among females, with the combined rate projected at approximately 12,054 per 100,000 person-years (Table 3). These projections suggest a sustained reduction in mortality burden, although the pace of decline appears more gradual compared with earlier years, and a segmented-regression analysis of the observed 2010–2023 series indicates this reflects a genuine change in trend around 2018 rather than a simple slowdown (Table S2 in S1 File). Projected declines are also observed in the late neonatal period, albeit at lower absolute levels. DALY rates among males are projected to decrease to approximately 291,645 per 100,000 person-years by 2030, while female rates are projected at 266,163 per 100,000 person-years, with the combined population reaching approximately 279,212 per 100,000 person-years (Table 3). Mortality trends follow a similar pattern. By 2030, late neonatal mortality is projected to decline to approximately 3,219 deaths per 100,000 person-years among males, compared with 2,936 per 100,000 person-years among females, with the combined rate estimated at approximately 3,081 per 100,000 person-years (Table 3). Across the twelve forecasted series (Deaths and DALYs, two age groups, three sex strata), the three forecasting approaches yielded projected 2030 estimates in broadly the same direction, but the ETS model produced materially different point estimates for several series (e.g., early-neonatal male deaths: log-linear 12,407 vs. ETS 19,524 per 100,000 person-years), and out-of-sample validation (three-year rolling-origin RMSE) did not show a consistent advantage for the log-linear model over the alternatives. The projected values above should therefore be interpreted as one plausible trend-based scenario rather than a precise forecast (see Table 3 for the full sensitivity comparison).

**Table 3:** Comparison of 2030 projections for all-cause neonatal deaths and DALYs in Kazakhstan across forecasting models, by neonatal period and sex.

Measure	Sex	Model	2030 estimate (95% PI)	AIC (in-sample)	BIC (in-sample)	RMSE (3-year holdout)
Early neonatal (0–6 days)						
Deaths	Male	Log-linear (primary)	12,407 (9,718−15,841)	−26.50	−24.60	2,817
		Linear	8,114 (1,769−14,460)	258.10	260.10	3,978
		ETS(A,N,N)	19,524 (10,567−28,481)	–	–	2,026
	Female	Log-linear (primary)	11,734 (7,827−17,592)	−12.30	−10.40	4,443
		Linear	10,150 (3,065−17,234)	261.20	263.10	5,127
		ETS(A,A,N)	22,814 (1,456−44,173)	–	–	1,891
	Both sexes	Log-linear (primary)	12,054 (8,850−16,417)	−19.90	−18.00	3,561
		Linear	9,095 (2,466−15,723)	259.40	261.30	4,518
		ETS(A,N,N)	18,831 (11,158−26,504)	–	–	1,911
DALYs	Male	Log-linear (primary)	1,117,929 (875,801−1,426,998)	−26.50	−24.60	253,599
		Linear	731,578 (160,448−1,302,708)	384.10	386.10	358,058
		ETS(A,A,N)	1,551,397 (103,079−2,999,715)	–	–	214,799
	Female	Log-linear (primary)	1,057,849 (706, 025 –1,584,994)	−12.40	−10.50	399,895
		Linear	915,181 (277,589−1,552,773)	387.20	389.10	461,422
		ETS(A,N,N)	1,631,477 (1,005,793−2,257,161)	–	–	199,948
	Both sexes	Log-linear (primary)	1,086,312 (797,867−1,479,037)	−19.90	−18.00	320,523
		Linear	820,012 (223,395−1,416,629)	385.40	387.30	406,645
		ETS(A,N,N)	1,696,586 (1,005,990−2,387,183)	–	–	185,163
Late neonatal (7–27 days)						
Deaths	Male	Log-linear (primary)	3,219 (2,745−3,776)	−38.40	−36.50	421
		Linear	3,072 (2,429−3,716)	194.10	196.00	413
		ETS(A,N,N)	3,613 (2,553−4,672)	–	–	361
	Female	Log-linear (primary)	2,936 (2,698−3,196)	−56.10	−54.20	145
		Linear	2,865 (2,555−3,174)	173.60	175.50	152
		ETS(A,N,N)	3,346 (2,713−3,979)	–	–	188
	Both sexes	Log-linear (primary)	3,081 (2,771−3,427)	−49.80	−47.90	227
		Linear	2,970 (2,566−3,374)	181.00	182.90	218
		ETS(A,N,N)	3,482 (2,665−4,299)	–	–	266
DALYs	Male	Log-linear (primary)	291,645 (249,034-341,547)	−38.70	−36.80	37,730
		Linear	278,263 (220,584−335,943)	319.90	321.90	37,021
		ETS(A,N,N)	327,478 (231,833−423,122)	–	–	32,460
	Female	Log-linear (primary)	266,163 (244,682−289,531)	−56.30	−54.40	13,072
		Linear	259,608 (231,746−287,470)	299.60	301.50	13,709
		ETS(A,N,N)	303,417 (244,378−362,456)	–	–	16,907
	Both sexes	Log-linear (primary)	279,212 (251,341−310,174)	−50.10	−48.20	20,330
		Linear	269,061 (232,849−305,272)	306.90	308.80	19,525
		ETS(A,N,N)	315,726 (241,299-390,153)	–	–	23,979

RMSE was calculated using 3-year rolling-origin out-of-sample forecasts for 2021−23 and is the scale-consistent metric for comparison across all three models. ETS model forms are denoted ETS(error, trend, seasonal); ETS(A,N,N) indicates additive error with no trend and no seasonal component (simple exponential smoothing), for which the 2030 point forecast equals the final smoothed level and therefore closely approximates the 2023 observed rate. Ten of the twelve series were fit with ETS(A,N,N); the remaining two (early-neonatal male DALYs and early-neonatal female Deaths) were fit with ETS(A,A,N) (additive trend, no seasonality), for which the 2030 forecast differs materially from the 2023 value.

Abbreviations: DALYs = disability-adjusted life-years. ETS = exponential smoothing. AIC = Akaike information criterion. BIC = Bayesian information criterion. RMSE = root mean square error. PI = prediction interval.

**Fig 5:**
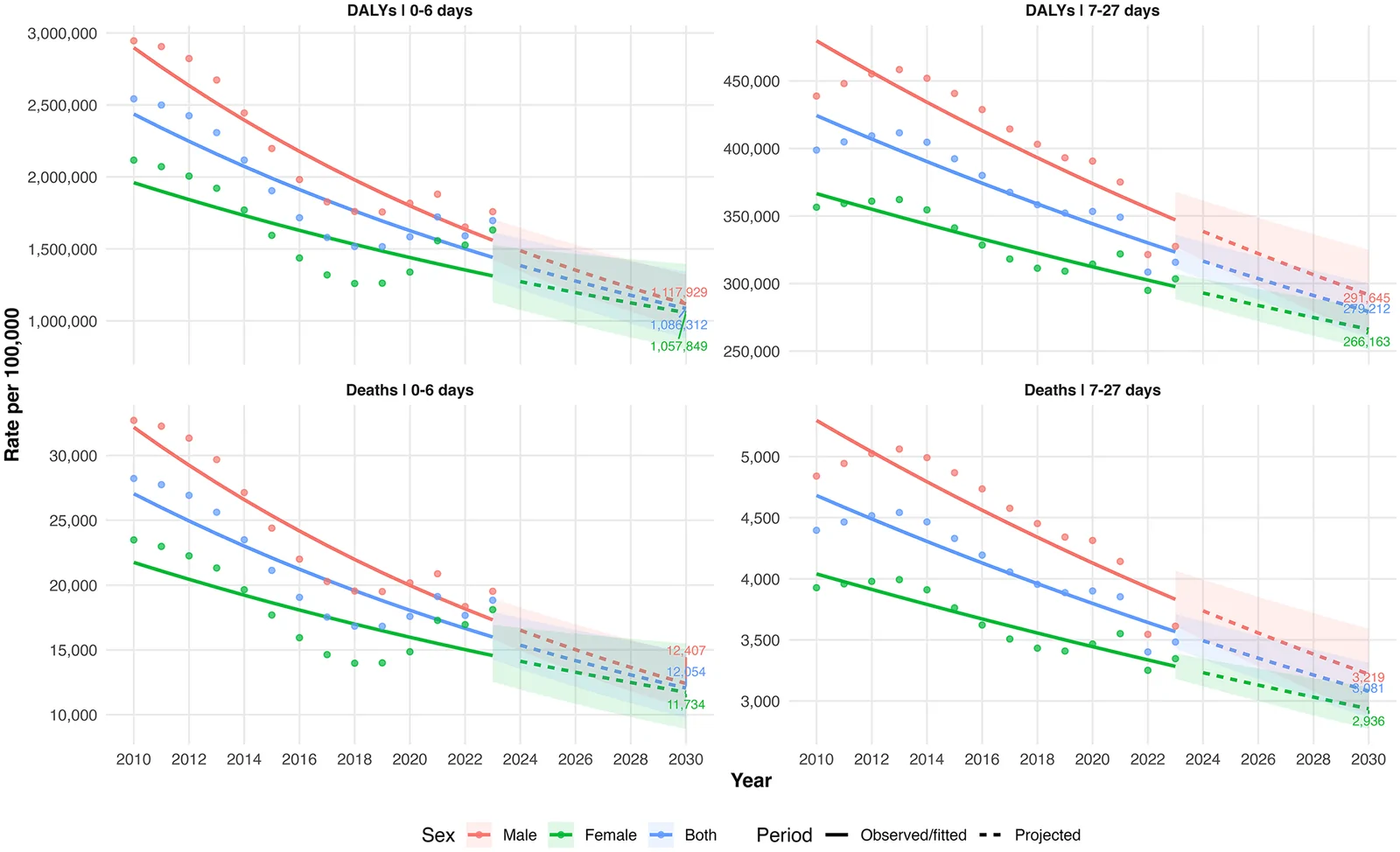
Projected neonatal mortality and DALY rates in Kazakhstan. Note. Primary log-linear model fitted to 2010-2023 GBD estimates in solid lines; dashed lines and shaded bands show 2024-2030 projections with 95% prediction intervals; points indicate observed estimates. GBD age-specific rates are expressed per 100,000 person-years within the specified neonatal age interval and are not mortality probabilities per live birth.

The national infant mortality rate showed broad temporal concordance with the GBD age-specific neonatal mortality series (Table 4). Across 2010–2023, the national series was strongly correlated with the GBD early-neonatal mortality rate in level (Pearson r = 0.96; Spearman ρ = 0.84) and was also positively correlated with the late-neonatal rate (Pearson r = 0.75; Spearman ρ = 0.83). Agreement was weaker for year-to-year changes: changes occurred in the same direction in 8 of 13 year-pairs for the early-neonatal series and 6 of 13 for the late-neonatal series, with first-difference correlations of r = 0.58 and r = 0.01, respectively. The national male infant mortality rate exceeded the corresponding all-population infant mortality rate throughout the study period; however, because a separate female national series was not included in the available comparison dataset, these data were not used to quantify or validate the magnitude of the male-to-female differences observed in the GBD estimates. These findings indicate broad long-term trend concordance, particularly for early-neonatal mortality, but limited short-term correspondence. Given differences in age scope, denominators, and partial overlap in underlying data sources, these comparisons should not be interpreted as external validation of the GBD estimates.

**Table 4:** Trend concordance between GBD age-specific neonatal mortality rates and national infant mortality rates in Kazakhstan, 2010–2023.

Comparison with national infant mortality	Pearson r, levels	Spearman ρ	Pearson r, first differences	Same direction
GBD early neonatal	0.96	0.84	0.58	8/13
GBD late neonatal	0.75	0.83	0.01	6/13

Note. GBD early-neonatal (0–6 days) and late-neonatal (7–27 days) mortality rates were compared separately with the national infant mortality rate because the two GBD age-specific rates have different person-time denominators and cannot be combined into a single 0–27-day rate. Pearson and Spearman coefficients describe concordance in annual levels; Pearson correlations of first differences assess concordance in year-to-year changes. “Same direction” indicates the number of consecutive year-pairs in which the GBD and national series changed in the same direction. These analyses assess temporal concordance rather than numerical agreement or external validation.

## 4. Discussion

This study provides a population-level assessment of neonatal mortality and disease burden in Kazakhstan, demonstrating substantial declines between 2010 and 2023 alongside persistent sex differences and marked concentration of mortality in the early neonatal period. Overall, the findings indicate substantial progress in reducing neonatal mortality, YLLs, YLDs, and DALYs over the study period. The decline was observed in both males and females and across both neonatal age groups. Mortality, YLL, and DALY point estimates remained higher among males across both neonatal subperiods, whereas YLD patterns differed by subperiod. Another important finding was the continued predominance of YLLs within total DALYs, indicating that premature mortality, rather than non-fatal disability, remains the principal contributor to neonatal burden in Kazakhstan. Although the primary log-linear projections extrapolate further declines, the segmented analysis identified a marked change in the observed early neonatal trajectory around 2018, with subsequent stalling and possible partial reversal. Together with the model dependence observed in the forecasting sensitivity analyses, this finding argues against interpreting continued improvement as assured.

The marked decline in neonatal burden observed in this study is consistent with global and regional evidence showing gradual improvements in neonatal survival over the past two decades, particularly in settings that have expanded access to facility-based childbirth, neonatal resuscitation, infection management, and care for preterm infants [9,10,19,20]. At the same time, our findings suggest that further reductions may become more difficult to achieve as mortality levels decline, since the remaining burden is often concentrated in more complex and less easily preventable conditions, including complications of prematurity, intrapartum-related events, and severe neonatal infections. In this context, the persistent dominance of YLLs is especially important, as it suggests that the greatest opportunities for further improvement lie in preventing fatal outcomes during the earliest and most vulnerable period of life.

The persistent male excess is consistent with the established male disadvantage in neonatal survival. Although the male-to-female rate ratio declined from approximately 1.35–1.40 in the early years to 1.08 by 2023, with recent uncertainty intervals approaching or including 1, the remaining disparity suggests persistent biological and perinatal vulnerability among male neonates despite substantial sex convergence. Male disadvantage in neonatal survival has been documented across diverse settings and is commonly attributed to biological vulnerability, including greater susceptibility to respiratory complications, prematurity-related morbidity, inflammatory responses, and adverse perinatal adaptation [21,22]. The narrowing of sex disparities documented here may reflect general improvements in neonatal and perinatal care that benefited both sexes. The continued, though narrowing, excess burden among males supports incorporating sex as a factor in population-level neonatal health surveillance and epidemiological risk stratification. We do not extend this to specific clinical recommendations such as NICU triage protocols, which would require individual-level clinical evidence (e.g., birth weight, gestational age, or admission-level outcome data) beyond the scope of this population-level GBD-based analysis. Rather than implying the need for sex-specific policy in isolation, these findings support the value of incorporating sex into broader, population-level risk-based neonatal care strategies.

From a public health perspective, this study not only documents national progress but also identifies persistent vulnerabilities. Although Kazakhstan’s neonatal mortality is relatively low by regional and international standards, direct comparison with the SDG 3.2.2 threshold of 12 deaths per 1,000 live births is not appropriate because the present analysis used the GBD age-specific person-year rate rather than the live-birth-based probability of death [23,24]. Segmented regression identified a change in early-neonatal mortality and DALY trends around 2018 (Davies’ test, p < 0.001 under the standard assumption of independent residuals; Table S2 in S1 File), with an average annual decline of approximately 7% before 2018 followed by a flat or slightly increasing trend of about 2% per year through 2023; because the underlying regression residuals showed significant autocorrelation (Table S3 in S1 File), this p-value should be interpreted as nominal rather than autocorrelation-robust (see Section 2.2). This pattern suggests a stalling, and possible partial reversal, of earlier progress rather than a continued but slower decline. Potential contributors, including changes in reporting, healthcare utilization, and the COVID-19 pandemic, warrant further investigation. Sustained reductions will therefore require continued improvement in antenatal, intrapartum, and immediate postnatal care, together with equitable implementation across the health system.

The observed national trends should be interpreted within the context of broader maternal and child health reforms in Kazakhstan that may plausibly have contributed to them, including the modernization of perinatal care, strengthening of primary healthcare, and efforts to improve continuity of care for mothers and infants, though none of these associations can be tested causally with the present ecological design. In the Kazakhstani context, rural healthcare modernization is a potentially relevant contextual factor given the country’s vast territory and the challenges of ensuring access to services in remote areas [25]. Expanded rural health infrastructure and service capacity may plausibly have contributed to more favorable conditions for maternal and neonatal care, though this cannot be established from the present data. The Universal Progressive Patronage Model is a further plausible contextual contributor, particularly through risk-stratified home visiting and earlier identification of family vulnerabilities [26]. In addition, implementation of the Compulsory Social Health Insurance system increased health expenditures and may have supported improvements in service availability and quality of care [27]. Because this study was based on secondary analysis of GBD estimates and used a descriptive design, it cannot determine the extent to which any of these reforms directly caused the observed declines; each is presented above as a plausible contextual contributor rather than a confirmed explanatory factor, and this limitation applies uniformly to the reforms discussed in this paragraph. These findings are consistent with previous international studies reporting higher neonatal mortality among males and greater vulnerability in the early neonatal period. However, evidence from Central Asia remains limited, and this study contributes region-specific data to the global literature. The projections presented in this study extrapolate historical 2010–2023 trends and do not incorporate future changes in health policy, healthcare financing, demographic structure, infectious disease outbreaks, or socioeconomic conditions, any of which could accelerate, slow, or reverse the observed trajectory; the 2030 estimates should therefore be interpreted as an extension of past patterns under continuity assumptions, not as an expected or most-likely future outcome.

Females exhibited modestly higher YLD rates than males in the early-neonatal period (Fig 3, Fig 4), a pattern also reported in some other GBD-based analyses, though its explanation remains uncertain and may partly reflect sex differences in the cause composition of non-fatal neonatal morbidity captured by GBD disability weights; this pattern was not present in the late-neonatal period, where YLD rates were nearly equal between sexes. Because YLDs contributed only a small fraction of total early-neonatal DALYs (well under 1%), the clinical and public-health significance of this sex difference is likely limited relative to the mortality-driven component of the male excess burden, and we present it descriptively rather than as a target for intervention. These findings have implications for neonatal health monitoring and underscore the importance of focusing on early neonatal survival. Continued improvements in neonatal outcomes are likely to depend on interventions targeting the immediate perinatal period. First, because fatal outcomes continue to account for the overwhelming share of neonatal burden, interventions should remain focused on preventing avoidable neonatal deaths, especially those related to preterm birth complications, intrapartum hypoxia, and severe infections. Second, the persistence of male disadvantage suggests that neonatal risk assessment frameworks may benefit from incorporating sex as one component of early population-level stratification. Third, the apparent stalling of progress after 2018 highlights the need to move beyond simple coverage expansion toward improvement in care quality, including timely identification of complications, neonatal resuscitation capacity, infection prevention and control, specialized care for vulnerable newborns, and strengthened follow-up during the first month of life. Finally, continued strengthening of primary healthcare and community-based support systems, including further expansion and optimization of the Universal Progressive Patronage Model, may help sustain gains by addressing maternal risk factors, improving care-seeking, and reinforcing postnatal monitoring, particularly among vulnerable families.

This study has several strengths. It provides a long-term national assessment using a standardized and internationally comparable data source, examines multiple dimensions of burden rather than mortality alone, stratifies findings by sex and neonatal age group, and extends the analysis to projected trends through 2030. These features enhance the relevance of the study for national planning and for situating Kazakhstan within the broader global neonatal health agenda.

This study also has several limitations. First, although GBD estimates are standardized and allow comparison across time and settings, the estimates are model-based and depend on the quality and completeness of underlying data sources; in settings with limited primary data, GBD estimates rely more heavily on statistical modeling and covariate-based imputation, which can increase uncertainty in ways not always fully reflected in the reported intervals. GBD estimates are also aggregated at the national level and cannot capture individual-level determinants of neonatal mortality (e.g., birth weight, gestational age, congenital anomalies, or intrapartum complications), limiting the clinical specificity of population-level findings such as these. Second, the study was ecological and descriptive; therefore, causal inferences regarding the impact of policies, reforms, or health system changes cannot be made. Third, the analysis was conducted at the national level and did not account for potential regional, rural-urban, socioeconomic, or ethnic inequalities that may shape neonatal outcomes within Kazakhstan. Fourth, the forecasting approach was based on historical trend extrapolation, fitted to GBD point estimates without propagating the underlying GBD uncertainty intervals (which were not available to the authors as posterior draws) and without correcting for the significant residual autocorrelation detected in most series; accordingly, the reported prediction intervals likely understate the true forecast uncertainty, and the projections should be interpreted as trend-based scenarios under continuity assumptions rather than precise forecasts, consistent with the forecasting sensitivity analysis, which did not show a consistent out-of-sample advantage for the primary log-linear model over the linear or ETS alternatives. This same residual autocorrelation also affects the EAPC 95% CIs and p-values (Table 2) and the Davies’ test p-value for the 2018 breakpoint (Table S2 in S1 File), both of which were computed under a standard assumption of independent residuals and should therefore be interpreted as nominal rather than autocorrelation-robust statistics (Section 2.2). Fifth, the approximate uncertainty intervals reported for the male-to-female rate ratios were computed under an independence assumption between the sex-specific GBD estimates, which is not strictly correct and may not fully capture the true correlation structure of the underlying GBD estimation process. Finally, the external comparison was used to assess trend concordance rather than one-to-one numerical agreement, given differences in age scope (0–27 days vs. 0–1 year), denominators, and the fact that GBD estimates are not fully independent of the national data sources used for comparison. Despite these limitations, the study offers a useful national perspective on progress, remaining challenges, and future priorities for neonatal survival in Kazakhstan.

## 5. Conclusion

Neonatal mortality and disease burden in Kazakhstan declined substantially between 2010 and 2023, with the greatest burden concentrated in the early neonatal period. Male point estimates for mortality, YLLs, and DALYs remained higher than female estimates throughout the observation period, although male-to-female differences narrowed substantially and uncertainty intervals for recent rate ratios included or approached unity. Because neonatal DALYs were overwhelmingly driven by YLLs, further reductions in burden depend principally on preventing early neonatal deaths. The observed stalling of early-neonatal progress after approximately 2018 warrants continued surveillance, while the model dependence of the 2030 projections indicates that these estimates should be interpreted as trend-based scenarios rather than precise forecasts.

## Supporting information

S1 File Supplementary tables. Table S1. Annual male-to-female rate ratios (RR) for neonatal mortality and burden, Kazakhstan, 2010–2023, with approximate 95% uncertainty intervals. Table S2. Segmented (broken-stick) regression of log(rate) on year, early neonatal period, both sexes combined, testing for a change in trend (Davies’ test for a non-constant slope). Table S3. Durbin–Watson (DW) and Breusch–Godfrey (BG, order 1) test statistics for residual autocorrelation in the log-linear models.
(DOCX)
