# Supplementary material for: Sex differences in early and late neonatal all-cause mortality and disease burden in Kazakhstan, 2010–2023: A population-based analysis of Global Burden of Disease estimates
Source: PLoS One. 2026 Oct 8;21(10):e0347911. doi: 10.1371/journal.pone.0347911 (PMC13649011; doi:10.1371/journal.pone.0347911)
Supplement: Supplementary file 1 [file pone.0347911.s001.docx]

**S1 File. Supplementary tables**

**Table S1. Annual male-to-female rate ratios (RR) for neonatal mortality and burden, Kazakhstan, 2010-2023, with approximate 95% uncertainty intervals**

| **Year** | **Deaths** **RR (95% UI)** | **DALYs** **RR (95% UI)** | **YLLs** **RR (95% UI)** | **YLDs** **RR (95% UI)** |
| --- | --- | --- | --- | --- |
| **Early neonatal (0-6 days)** | | | | |
| 2010 | 1.39 (1.12-1.74) | 1.39 (1.12-1.73) | 1.39 (1.12-1.74) | 0.87 (0.55-1.38) |
| 2011 | 1.40 (1.12-1.75) | 1.40 (1.12-1.75) | 1.40 (1.12-1.75) | 0.87 (0.54-1.39) |
| 2012 | 1.41 (1.15-1.73) | 1.41 (1.15-1.73) | 1.41 (1.15-1.73) | 0.86 (0.54-1.39) |
| 2013 | 1.39 (1.15-1.69) | 1.39 (1.15-1.68) | 1.39 (1.15-1.69) | 0.86 (0.53-1.39) |
| 2014 | 1.38 (1.15-1.66) | 1.38 (1.15-1.66) | 1.38 (1.15-1.66) | 0.85 (0.53-1.38) |
| 2015 | 1.38 (1.15-1.66) | 1.38 (1.15-1.66) | 1.38 (1.15-1.66) | 0.85 (0.52-1.39) |
| 2016 | 1.38 (1.15-1.66) | 1.38 (1.15-1.66) | 1.38 (1.15-1.66) | 0.84 (0.52-1.37) |
| 2017 | 1.39 (1.15-1.67) | 1.38 (1.15-1.67) | 1.39 (1.15-1.67) | 0.83 (0.50-1.38) |
| 2018 | 1.40 (1.17-1.68) | 1.40 (1.16-1.68) | 1.40 (1.17-1.68) | 0.83 (0.49-1.39) |
| 2019 | 1.39 (1.19-1.62) | 1.39 (1.19-1.62) | 1.39 (1.19-1.62) | 0.82 (0.48-1.41) |
| 2020 | 1.36 (1.20-1.53) | 1.36 (1.20-1.53) | 1.36 (1.20-1.53) | 0.82 (0.48-1.39) |
| 2021 | 1.21 (1.06-1.37) | 1.21 (1.06-1.37) | 1.21 (1.06-1.37) | 0.82 (0.49-1.39) |
| 2022 | 1.08 (0.82-1.42) | 1.08 (0.82-1.42) | 1.08 (0.82-1.42) | 0.83 (0.49-1.41) |
| 2023 | 1.08 (0.78-1.49) | 1.08 (0.78-1.48) | 1.08 (0.78-1.49) | 0.83 (0.49-1.38) |
| **Late neonatal (7-27 days)** | | | | |
| 2010 | 1.23 (0.97-1.57) | 1.23 (0.97-1.56) | 1.23 (0.97-1.57) | 1.04 (0.63-1.70) |
| 2011 | 1.25 (0.99-1.57) | 1.25 (0.99-1.57) | 1.25 (0.99-1.57) | 1.03 (0.63-1.71) |
| 2012 | 1.26 (1.01-1.58) | 1.26 (1.01-1.57) | 1.26 (1.01-1.58) | 1.03 (0.62-1.72) |
| 2013 | 1.27 (1.02-1.57) | 1.27 (1.02-1.57) | 1.27 (1.02-1.57) | 1.03 (0.61-1.74) |
| 2014 | 1.28 (1.03-1.58) | 1.27 (1.03-1.57) | 1.28 (1.03-1.58) | 1.02 (0.60-1.73) |
| 2015 | 1.29 (1.05-1.59) | 1.29 (1.05-1.58) | 1.29 (1.05-1.59) | 1.02 (0.60-1.72) |
| 2016 | 1.31 (1.07-1.60) | 1.31 (1.07-1.60) | 1.31 (1.07-1.60) | 1.01 (0.60-1.71) |
| 2017 | 1.31 (1.07-1.59) | 1.30 (1.07-1.59) | 1.31 (1.07-1.59) | 1.01 (0.59-1.72) |
| 2018 | 1.30 (1.07-1.57) | 1.29 (1.07-1.56) | 1.30 (1.07-1.57) | 1.00 (0.59-1.71) |
| 2019 | 1.27 (1.07-1.52) | 1.27 (1.07-1.52) | 1.27 (1.07-1.52) | 1.00 (0.59-1.72) |
| 2020 | 1.24 (1.07-1.44) | 1.24 (1.07-1.44) | 1.24 (1.07-1.44) | 1.00 (0.57-1.75) |
| 2021 | 1.17 (0.99-1.37) | 1.17 (0.99-1.37) | 1.17 (0.99-1.37) | 1.02 (0.60-1.73) |
| 2022 | 1.09 (0.80-1.49) | 1.09 (0.80-1.48) | 1.09 (0.80-1.49) | 1.02 (0.61-1.73) |
| 2023 | 1.08 (0.75-1.56) | 1.08 (0.75-1.56) | 1.08 (0.75-1.56) | 1.03 (0.61-1.72) |

Note. Approximate UIs were computed by the delta method [SE of log-RR = sqrt(SE(log Male)^2 + SE(log Female)^2)] under an assumption of independence between the sex-specific GBD estimates. Because male and female GBD estimates for the same location, cause, and year share underlying data inputs and modeling structure, the independence assumption is not strictly correct. Because their joint covariance was unavailable, the direction and magnitude of any approximation error cannot be determined; accordingly, these intervals should be regarded as approximate. Posterior draw-level data were not available to compute draw-based RR uncertainty.

Abbreviations: DALYs=disability-adjusted life-years. YLLs=years of life lost. YLDs=years lived with disability. RR=rate ratio. UI=uncertainty interval.

**Table S2. Segmented (broken-stick) regression of log(rate) on year, early neonatal period, both sexes combined, testing for a change in trend (Davies' test for a non-constant slope)**

| **Measure** | **Breakpoint year** | **Slope before** | **Slope after** | **Davies' test *p* value** |
| --- | --- | --- | --- | --- |
| Deaths | 2018 | -0.0722 | 0.023 | 0.000129 |
| DALYs | 2018 | -0.0721 | 0.023 | 0.000129 |

Note. Slopes are on the log scale (approximate annual proportional change). A significant Davies' test (p<0.05) indicates that the single-slope log-linear model is misspecified and that the trend changed at the identified breakpoint. The results show a statistically significant breakpoint around 2018, with a shift from a declining trend (2010-2018) to a non-declining or slightly increasing trend (2018-2023), supporting a genuine change in trajectory (stalling), rather than only a change in the rate of decline.

**Table S3. Durbin-Watson (DW) and Breusch-Godfrey (BG, order 1) test statistics for residual autocorrelation in the log-linear models**

| **Measure** | **Sex** | **DW statistic** | **DW *p* value** | **BG *p* value** |
| --- | --- | --- | --- | --- |
| **Early neonatal (0-6 days)** | | | | |
| Deaths | Male | 0.56 | 7.08e-05 | 0.007 |
|  | Female | 0.31 | 9.65e-08 | 0.001 |
|  | Both sexes | 0.39 | 1.62e-06 | 0.002 |
| DALYs | Male | 0.56 | 7.05e-05 | 0.007 |
|  | Female | 0.31 | 9.63e-08 | 0.001 |
|  | Both sexes | 0.39 | 1.61e-06 | 0.002 |
| **Late neonatal (7-27 days)** | | | | |
| Deaths | Male | 0.72 | 5.69e-04 | 0.058 |
|  | Female | 1.31 | 0.042 | 0.273 |
|  | Both sexes | 1.11 | 0.014 | 0.255 |
| DALYs | Male | 0.72 | 5.86e-04 | 0.058 |
|  | Female | 1.31 | 0.041 | 0.271 |
|  | Both sexes | 1.12 | 0.015 | 0.259 |

Note. DW values well below 2 with small p values indicate significant positive autocorrelation in most series, confirming that annual GBD estimates behave as a time series rather than independent observations. Standard OLS prediction intervals assume independent errors and therefore likely understate the true uncertainty of the 2030 projections; this is stated explicitly as a limitation in the manuscript.

Abbreviations: DW=Durbin-Watson. BG=Breusch-Godfrey. OLS=ordinary least squares.
